# Maternal Arterial Stiffness in Women Who Subsequently Develop Pre-Eclampsia

**DOI:** 10.1371/journal.pone.0018703

**Published:** 2011-05-03

**Authors:** Makrina D. Savvidou, Christina Kaihura, James M. Anderson, Kypros H. Nicolaides

**Affiliations:** 1 Department of Maternal Fetal Medicine, Imperial College School of Medicine, Chelsea and Westminster Hospital, London, United Kingdom; 2 Harris Birthright Research Centre for Fetal Medicine, King's College Hospital, London, United Kingdom; New York University School of Medicine, United States of America

## Abstract

**Background/Objectives:**

Pre-eclampsia (PE) is associated with profound changes in the maternal cardiovascular system. The aim of the present study was to assess whether alterations in the maternal arterial stiffness precede the onset of PE in at risk women.

**Methodology/Principal Findings:**

This was a cross sectional study involving 70 pregnant women with normal and 70 women with abnormal uterine artery Doppler examination at 22–24 weeks of gestation. All women had their arterial stiffness (augmentation index and pulse wave velocity of the carotid-femoral and carotid-radial parts of the arterial tree) assessed by applanation tonometry in the second trimester of pregnancy, at the time of the uterine artery Doppler imaging. Among the 140 women participating in the study 29 developed PE (PE group) and 111 did not (non-PE group). Compared to the non-PE group, women that developed PE had higher central systolic (94.9±8.6 mmHg vs 104.3±11.1 mmHg; p = <0.01) and diastolic (64.0±6.0 vs 72.4±9.1; p<0.01) blood pressures. All the arterial stiffness indices were adjusted for possible confounders and expressed as multiples of the median (MoM) of the non-PE group. The adjusted median augmentation index was similar between the two groups (p = 0.84). The adjusted median pulse wave velocities were higher in the PE group compared to the non-PE group (carotid-femoral: 1.10±0.14 MoMs vs 0.99±0.11 MoMs; p<0.01 and carotid-radial: 1.08±0.12 MoMs vs 1.0±0.11 MoMs; p<0.01).

**Conclusions/Significance:**

Increased maternal arterial stiffness, as assessed by pulse wave velocity, predates the development of PE in at risk women.

## Introduction

Pre-eclampsia (PE), which affects 2% of pregnancies, is one of the leading causes of maternal and perinatal mortality and morbidity [1]. The underlying pathophysiological mechanism is thought to be impaired trophoblastic invasion of the maternal spiral arteries with consequent placental hypoperfusion and hypoxia [2], [3]. The abnormal trophoblastic invasion can be detected non-invasively by Doppler examination of the uterine arteries which, at mid-gestation, show evidence of high resistance in 77% of cases affected by early onset severe PE [4], [5].

In pregnancies with PE, there is some evidence that in addition to the vascular changes in the uteroplacental unit there is a generalized increase in maternal arterial stiffness [6]–[11]. Non-invasive assessment of arterial stiffness is possible by the simple, validated and reproducible technique of applanation tonometry with which central blood pressures, arterial wave reflection (augmentation index; AIx) and pulse wave velocity (PWV) of different parts of the arterial tree can be studied [12]–[15]. Arterial stiffness has been shown to be an independent predictor of cardiovascular events and mortality in healthy non-pregnant subjects [16]. Six studies, utilizing applanation tonometry in pregnant women with established PE, have reported inconsistent results regarding maternal arterial stiffness but the majority of them support the concept of increased stiffness [6]–[11]. In women with PE, compared to normotensive controls, PWV and AIx were both increased in one study [6] whereas in a further study, none of them was increased following adjustment for possible confounders [7]. Kaihura et al reported that only PWV, but not AIx, was increased [8] whereas the last three studies only assessed AIx and this was found to be elevated [9]–[11].

In the current study, we have used Doppler examination of the uterine arteries in the second trimester of pregnancy in order to identify women at risk of PE. We sought to investigate whether altered maternal arterial stiffness at mid-pregnancy, as assessed by applanation tonometry, precedes the onset of PE.

## Materials and Methods

### Ethics statement

The study was approved by the Bexley and Greenwich Local Research Ethics Committee and all subjects gave written informed consent prior to participation in the study.

All women attending for routine antenatal care at King's College Hospital have color Doppler examination of their uterine arteries at 22–24 weeks of gestation. Trans-vaginal sonography is performed, the left and right uterine arteries are identified by color flow mapping, pulse wave Doppler is used to measure the pulsatility index (PI) in each vessel and the average PI of the two arteries (mean PI) is recorded [17]. In this study, at the time of the uterine artery Doppler imaging we performed applanation tonometry in 70 consecutive women with mean uterine artery PI above the 95^th^ percentile of a reference group (mean PI≥1.6) and 70 controls with mean PI below the 95^th^ percentile [17]. All 140 women had singleton pregnancies, were healthy, on no cardiovascular medications, had no personal history of hypertension or family history of premature cardiovascular disease and had appropriately grown fetuses for the gestation at the time of scanning. Maternal age, racial origin, smoking status, parity and body mass index (BMI) were recorded at recruitment.

### Wave reflection and arterial stiffness measurements

Peripheral blood pressure (BP) was measured in the right arm using an ambulatory blood pressure monitor (Microlife Medical 90207, WA, US), which has been validated for use in pregnancy [18]. Systolic and diastolic BP were measured twice and averaged.

**Figure 1:**
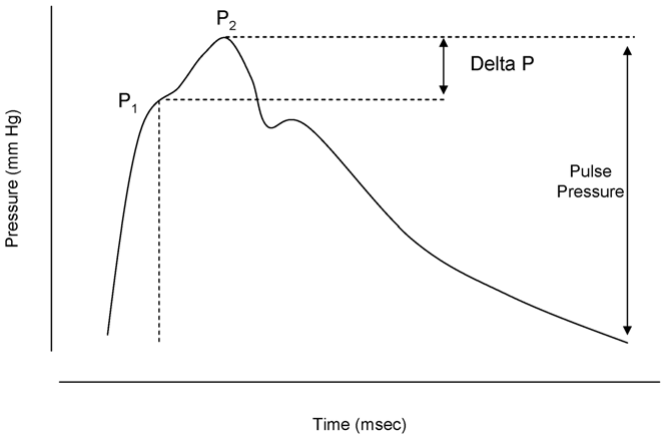
Typical ascending aortic waveform. Typical ascending waveform, showing two systolic peaks (P1 and P2). Augmentation index is calculated as the difference between P2 and P1, expressed as percentage of pulse pressure.

Each heartbeat generates a pulse wave that travels away from the heart and is reflected back at the areas of high resistance. The reflected wave travels back towards the heart and meets the advancing wave, augmenting its height. Generally, the reflected wave reaches the aorta during diastole, enhancing the cardiac perfusion. When arterial stiffness in increased, the arterial pulse wave travels faster, so the reflected wave reaches advancing wave in the systole, resulting in significant augmentation of the systolic peak. This can be measured as increased augmentation index (Figure 1). Interrogation of the radial artery waveform (pulse wave analysis of the radial artery) can provide information on augmentation index and the central, aortic haemodynamics [12], [13], [15]. Radial artery waveforms were obtained with a high-fidelity micromanometer (SPC-301; Millar Instruments, Houston, Tex) from the wrist, and a corresponding central waveform was generated with a validated transfer function (Sphygmocor; AtCor Medical, Sydney, Australia) [12], [13], [15]. Augmentation index, a composite measure of systemic arterial stiffness and wave-reflection amplitude, and central systolic, diastolic, pulse pressure were determined with the integrated software. Due to the linear correlation between AIx and heart rate (HR), AIx was standardized to a heart rate of 75 beats per minute (bpm); AIx-75. Information on aortic Tr (time between the start of the systolic curve and the inflection point) was also given. Mean arterial pressure (MAP) was obtained by integration of the waveform. Aortic (carotid-femoral) and brachial (carotid-radial) PWV were also measured, as previously described [14], [15]. All measurements were performed after a period of rest of at least 10 minutes in a left lateral position in order to avoid vena cava compression by the uterus. All measurements were made in duplicate and mean values were used for the subsequent analyses.

### Definition of outcome

The diagnosis of PE was made according to the criteria of the International Society for the Study of Hypertension in Pregnancy [19]. Under this classification, PE was defined as diastolic BP of at least 110 mmHg on one occasion or diastolic BP of at least 90 mmHg on two consecutive occasions more than four hours apart, in combination with proteinuria (≥300 mg total protein in a 24-hour urine collection or, if this was not available, ≥+2 proteinuria by dipstick analysis on two consecutive occasions at least four hours apart) developing after 20 weeks of gestation in previously normotensive women.

### Statistical analysis

Normality of the distribution of the data was examined with the Kolmogorov-Smirnov test. For those parameters that were not normally distributed logarithmic transformation was performed. Data were expressed as mean ± standard deviation or as median and interquartile range (IQR) for normally and non-normally distributed data, respectively. Comparisons between groups were performed using t-test, Mann-Whitney or chi-square (χ^2^) for numerical and categorical data, respectively. In order to compare the values of AIx-75 and PWV between those who subsequently developed PE (PE group) and those who did not (non-PE group) adjusting for variables that are known determinants of them, the following three steps were taken: firstly, in the non-PE group multiple regression analysis was used to determine which factors among the maternal demographic and vascular characteristics (maternal age, racial origin, smoking status, parity, BMI, mean uterine artery PI on Doppler examination, HR, MAP and aortic Tr) were significant predictors of AIx-75 and PWV (carotid-radial and carotid-femoral); secondly, in each woman the measured values of AIx-75 and PWVs were expressed as multiples of the median (MoM) of the non-PE group and thirdly, the median MoM values of AIx-75 and PWVs in the PE and non-PE groups were compared. Power analysis indicated that a sample of 11 women with PE and similar number of controls would have an 80% power with an alpha .05 (2-tails) for the detection of a mean difference of 1.7 m/sec in the PWV (carotid-femoral) between the groups. The effect size was estimated from previous publications [6], [8]. The statistical analyses were performed using the Statistical Package for Social Sciences (Version 12.0).

## Results

Recordings were successfully obtained from all 140 women and they all tolerated the studies well. One hundred and eleven women remained normotensive (non-PE group) whereas, in 27 of the cases in the high PI group (38.5%) and in 2 of those with normal PI (2.8%), the pregnancies were complicated by PE. The demographic characteristics of the PE and non-PE groups are compared in Table 1. The women in the PE group were more likely to be black, heavier, have previous pregnancy affected by PE and more likely to deliver smaller neonates, earlier.

**Table 1:** Maternal demographic and pregnancy characteristics of the non-preeclampsia and preeclampsia groups.

Parameter	Non-preeclampsian = 111	Preeclampsian = 29	*P* value
Maternal age (yrs)	30.8±6.3	29.4±5.7	0.57
Racial origin			<0.01
White, n (%)	61 (55.0)	4 (13.8)	
Black, n (%)	46 (41.4)	20 (69.0)	
Others, n (%)	4 (3.6)	5 (17.2)	
Smoking, n (%)	21 (18.9)	7 (24.1)	0.73
Nulliparity, n (%)	60 (54.1)	14 (48.3)	0.57
Maternal height (m)	1.6±0.05	1.6±0.05	0.80
Maternal weight (kg)	70.9±10.7	78.3±12.9	<0.01
Body mass index (kg/m^2^)	26.7±4.1	29.4±4.4	<0.01
Previous history of pre-eclampsia, n (%)	2 (1.8)	4 (13.8)	<0.01
Gestational age at entry (days)	161 (160–166)	163 (161–169)	0.04
Gestational age at delivery (days)	279 (270–285)	245 (220–267)	0.01
Birth weight (gr)	3216 (2781–3540)	2190 (1363–2779)	.01

Values are given as mean ± standard deviation or as median (interquartile range) for normally and not normally distributed data respectively.

The maternal haemodynamic and vascular characteristics according to the Doppler examination of the uterine arteries are given in Table 2. Pulse wave velocity, but not AIx-75, was significantly increased in women with ultrasonographic evidence of impaired placentation compared to women with normal placentation. In the non-PE group multiple regression analyses demonstrated that AIx-75 and PWV (carotid-femoral and carotid-radial) were significantly affected by maternal demographic and vascular characteristics as follows:

**Table 2:** Maternal haemodynamic and vascular characteristics of the study population according to the uterine artery Doppler examination.

Parameter	Normal uterine arteryDoppler examinationn = 70	Abnormal uterine arteryDoppler examinationn = 70	*P* value
Mean uterine artery PI	0.99 (0.83–1.12)	1.82 (1.73–2.03)	<0.01
Heart rate (bpm)	76.9±9.9	74.9±10.0	0.22
Heart cycle (ms)	792.2±102.6	815.1±107.9	0.20
Ejection duration (msec)	326.9±22.1	324.6±23.1	0.53
Diastole time (msec)	465.2±91.1	490.4±91.3	0.10
Peripheral systolic blood pressure (mmHg)	115.4±8.7	115.8±10.8	0.79
Peripheral diastolic blood pressure (mmHg)	67.0±7.0	66.8±8.0	0.83
Mean arterial pressure (mmHg)	77.7 (74.3–81.5)	79.2 (74.8–86.2)	0.25
Peripheral pulse pressure (mmHg)	48.3±6.7	49.0±8.6	0.59
Central systolic blood pressure (mmHg)	95.8±8.5	98.0±11.1	0.19
Central diastolic blood pressure (mmHg)	65.1±5.8	66.4±8.9	0.32
Central pulse pressure (mmHg)	30.6±6.0	31.5±5.9	0.36
Aortic Tr (msec)	157.7 (147.5–177.6)	152.7 (146.3–170.2)	0.35
Augmentation Index (%) at 75 bpm	5.2 (−5.1–13.0)	5.7 (−3.6–12.1)	0.44
Pulse wave velocity (carotid-femoral) (m/sec)	5.0±0.6	5.5±0.9	<0.01
Pulse wave velocity (carotid-radial) (m/sec)	7.4±1.0	7.9±1.0	0.01

Values are given as mean ± standard deviation or as median (interquartile range) for normally and not normally distributed data respectively.

AIx-75 expected = −51.72+0.61×maternal age in years+95.21×log MAP−65.07×log aortic Tr; R^2^ = 0.42, p<0.0001. Maternal racial origin, smoking status, parity, BMI and mean uterine artery PI were not significant predictors of AIx-75.

PWV (carotid-femoral) expected = −8.40+0.05×maternal age in years+4.91×log MAP+0.02×HR+0.28×mean uterine artery PI; R^2^ = 0.41, p<0.0001. Maternal racial origin, smoking status, parity and BMI were not significant predictors of PWV (carotid-femoral).

PWV (carotid-radial) expected = −2.81+0.04×maternal age in years+5.57×log MAP+(−1.86 if the racial origin was White, −1.33 if Black, 0 if other); R^2^ = 0.23, p<0.0001. Maternal smoking status, parity, BMI and mean uterine artery PI were not significant predictors of PWV (carotid-radial).

### Hemodynamic and vascular parameters in the PE group

**Figure 2:**
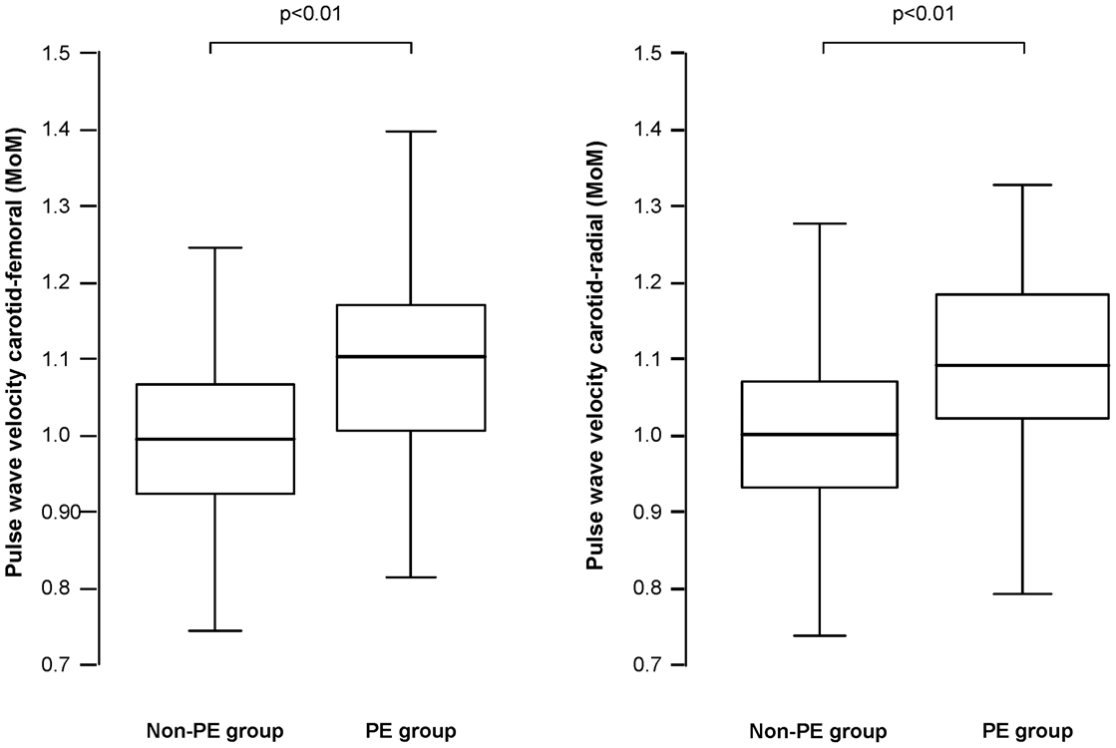
Pulse wave velocity in the study populations. Box and whisker plots comparing the maternal pulse wave velocity of the carotid-femoral and carotid-radial parts of the arterial tree, expressed as multiples of the median (MoM) in women who subsequently developed pre-eclampsia (PE group) and in the non-preeclampsia group (Non-PE group). Boxes represent inter-quartile range, where the line represents the median. Whiskers at top and bottom of the box represent the highest and lowest values.

The hemodynamic and vascular parameters of the study groups according to the outcome of pregnancy are given in Table S1. The median adjusted AIx-75 was not significantly different between the PE and non-PE groups. Conversely, the peripheral and central, systolic, diastolic and mean BP and adjusted PWV (carotid-femoral and carotid-radial) were increased in the PE group (Figure 2). Within the PE group, there was no statistical significant difference in the adjusted PWV's between the women with normal (n = 2) and those with abnormal (n = 27) uterine artery Doppler examination (p>0.05). In the PE group, there was no significant association between gestation at delivery and peripheral systolic BP (p = 0.41), peripheral diastolic BP (p = 0.15), central systolic BP (p = 0.22), central diastolic BP (p = 0.369) and MAP (p = 0.26). There was no statistically significant difference in the adjusted median PWV (carotid-femoral) values between women with early onset PE, requiring delivery prior to 34 weeks, compared to those with late onset disease (1.13±0.14 MoMs vs 1.07±0.14 MoMs; p = 0.28) or between those with concurrent fetal growth restriction and those without (p = 0.48).

## Discussion

The findings of this study demonstrate that in women destined to develop PE, during the second-trimester of pregnancy there is an increase in maternal arterial stiffness as assessed by PWV of the carotid-femoral and carotid-radial parts of the arterial tree. The magnitude of the PWV increase of about 17% is similar to that reported in women with established PE [6], [8] and although small, is likely to be clinically significant considering the fact that aortic PWV increases by only ∼6% per decade in healthy individuals [20].

Overall, women with impaired placentation, as detected by Doppler examination of the uterine arteries, had increased arterial stiffness (PWV) suggesting that women at risk of developing PE have a high resistance circulation affecting different vascular beds including the fetoplacental unit and the maternal conduit arteries. It is likely that other, multiple factors such as maternal genetic susceptibility will eventually determine which women will develop PE. Mean uterine artery PI was a significant independent predictor of PWV but despite that, PWV was still increased in women who subsequently developed PE implying that this vascular index provides additional information regarding the maternal cardiovascular adaptation to pregnancy over and above the Doppler examination of the uterine arteries.

The increased maternal arterial stiffness in women destined to develop PE may be related to the aberrant maternal physiological and biochemical adaptation to pregnancy that these women demonstrate. Maternal endothelial dysfunction, as assessed by flow-mediated dilatation of the brachial artery [21], increased levels of asymmetric dimethyl-arginine, an endogenous inhibitor of nitric oxide synthase [21], elevated concentrations of homocysteine and marked insulin resistance are all features of the pre-clinical state of PE [22], [23] and have also been shown to be associated with increased arterial stiffness [24], [25]. However, it is uncertain whether all these factors including maternal arterial stiffness are the cause or the phenotypic expression of the already existing underlying pathophysiological mechanisms of PE. Only studies in women prior, during and following pregnancy will be able to address this question.

Previous studies have shown that in non-pregnant populations increased PWV is predictive of cardiovascular mortality [16]. Furthermore, studies in women with established and previous history of PE have shown increased maternal arterial stiffness, as assessed by PWV [6]–[11], [26]. Our study, which is the first to assess maternal PWV prior to the clinical manifestation of PE, is also consistent with the above findings. Consequently, it could be hypothesised that increased arterial stiffness, as assessed by PWV, provides a plausible link between the development of PE in the index pregnancy and the increased propensity to cardiovascular events that these women experience later on in life [27]. Only studies assessing maternal arterial stiffness prior to, during and many years following a pregnancy complicated by PE can confirm the above concept.

In addition to increased arterial stiffness, women who subsequently developed PE demonstrated increased peripheral and central BP. Studies in non-pregnant hypertensive patients have shown that central and peripheral BP are not synonymous and antihypertensive agents can exert differential effects on the two types of BP [28]. In patients with end-stage renal disease, central aortic pulse pressure was of greater predictive value for cardiovascular outcomes than brachial pulse pressure [28], [29]. Previous studies assessing peripheral BP have reported that in women destined to develop PE, the BP is higher than in the non-PE group both during the second but also in the first-trimester of pregnancy [30]. It would be interesting to investigate the extent to which the prediction of PE can be improved by the measurement of central rather than peripheral BP.

In contrast to PWV, there were no significant differences in the AIx between the PE and non-PE groups. This is compatible with the results of our previous study in women with established PE where there was an increase in PWV but not in AIx [8]. However, it is in contrast with other studies that suggested that AIx is elevated in women with established PE [6], [9]–[11] and one study that suggested that AIx could be used as a first trimester predictor of PE [31]. Augmentation index provides an indirect measure of arterial stiffness and in both healthy individuals and in those with a disease such as hypercholesterolemia [32] and essential hypertension [33], there is usually an association between PWV and AIx. However, AIx depends on the intensity of the reflected wave and as such it will depend on the diameter and elasticity of the small muscular arteries/arterioles at the major sites of pressure wave reflection. Therefore, alterations in muscular smooth muscle tone affecting mainly the small muscular arteries but not the elastic aorta might influence reflected wave intensity and hence AIx independently of PWV. In accordance to this, administration of vasoactive substances will affect AIx and PWV differently [34]. Furthermore, the dissociation between PWV and AIx observed in our study has also been described in individuals with metabolic syndrome [35], a condition that is also present in a number of women who develop PE [23].

The aim of the current study was to investigate the maternal cardiovascular adaptation and in particular arterial stiffness in women destined to develop PE and not to assess whether arterial stiffness indices could be used as predictors of PE development. Therefore, we did not attempt to create predictive models and receiver-operating characteristics curve analysis. Furthermore, this was a cross-sectional study and as such we cannot comment on the longitudinal changes of maternal arterial stiffness during pregnancy complicated by PE. However, our results should encourage further research, involving larger number of women, to establish the predictive value of PWV in PE development and its use in patient's management.

The study demonstrated significant maternal hemodynamic/arterial stiffness differences between women destined to develop PE and those did not. The extent to which arterial stiffness is useful in screening for PE in unselected and high risk populations remains to be determined.

## Supporting Information

Table S1 **Haemodynamic and vascular parameters of the non-preeclampsia and preeclampsia groups.**
(DOC)
