# Supplementary material for: Maternal Arterial Stiffness in Women Who Subsequently Develop Pre-Eclampsia
Source: PLoS One. 2011 May 3;6(5):e18703. doi: 10.1371/journal.pone.0018703 (PMC3086903; doi:10.1371/journal.pone.0018703)
Supplement: Table S1 — Haemodynamic and vascular parameters of the non-preeclampsia and preeclampsia groups. (DOC) [file pone.0018703.s001.doc]

**Table S1.** **Haemodynamic and vascular parameters of the non-preeclampsia and preeclampsia groups.**

| **Parameter** | **Non-preeclampsia**  **n=111** | **Preeclampsia**  **n=29** | ***P* value** |
| --- | --- | --- | --- |
| Mean uterine artery pulsatility index | 1.16 (0.91-1.74) | 1.83 (1.75-2.1) | <0.01 |
| Heart rate (bpm) | 75.1 ± 9.5 | 78.8 ± 11.2 | 0.08 |
| Heart cycle (ms) | 810.0 ± 101.1 | 777.2 ± 119.3 | 0.13 |
| Ejection duration (msec) | 327.6 ± 22.3 | 318.7 ± 22.4 | 0.06 |
| Diastole time (msec) | 482.9 ± 89.1 | 458.5 ± 100.4 | 0.20 |
| Peripheral systolic blood pressure (mmHg) | 114.3 ± 8.3 | 120.8 ± 13.0 | 0.01 |
| Peripheral diastolic blood pressure (mmHg) | 65.3 ± 6.7 | 73.1 ± 7.3 | <0.01 |
| Mean arterial pressure (mmHg) | 77.5 (73.5- 81.0) | 86.0 (81.0-94.7) | <0.01 |
| Peripheral pulse pressure (mmHg) | 49.0 ± 7.0 | 47.6 ± 10.2 | 0.62 |
| Central systolic blood pressure (mmHg) | 94.9 ± 8.6 | 104.3 ± 11.1 | <0.01 |
| Central diastolic blood pressure (mmHg) | 64.0 ± 6.0 | 72.4 ± 9.1 | <0.01 |
| Central pulse pressure (mmHg) | 30.9 ± 5.8 | 31.8 ± 6.4 | 0.45 |
| Aortic Tr (msec) | 157.5 (147.4-177.5) | 149.0 (143.7-163.7) | 0.02 |
| Augmentation Index at 75 bpm, Raw values (%) | 4.5 (-5-11.5) | 8.0 (3.0-18.7) | 0.02 |
| Augmentation Index at 75 bpm, Adjusted values (MoM’s)* | 1.0 (0.01-1.93) | 0.80 (0.42-2.01) | 0.84 |
| Pulse wave velocity (carotid-femoral), Raw values (m/sec) | 5.0 ± 0.7 | 6.0 ± 0.8 | <0.01 |
| Pulse wave velocity (carotid-femoral), Adjusted values (MoM’s) ** | 0.99 ± 0.11 | 1.10 ± 0.14 | <0.01 |
| Pulse wave velocity (carotid-radial), Raw values (m/sec) | 7.4 ± 0.9 | 8.7 ± 0.9 | <0.01 |
| Pulse wave velocity (carotid-radial), Adjusted values (MoM’s) † | 1.00 ± 0.11 | 1.08 ± 0.12 | <0.01 |

Values are given as mean ± standard deviation or as median (interquartile range) for normally and not normally distributed data respectively.

bpm: beats per minute

MoM: multiple of the median

* Values of augmentation Index at 75bpm adjusted for maternal age and mean blood pressure

** Values of pulse wave velocity (carotid-femoral) adjusted for maternal age, mean blood pressure, heart rate and mean uterine artery pulsatility index

† Values of pulse wave velocity (carotid-radial) adjusted for maternal age, mean blood pressure and racial origin
